# Renal Sensory Activity Regulates the γ-Aminobutyric Acidergic Inputs to the Paraventricular Nucleus of the Hypothalamus in Goldblatt Hypertension

**DOI:** 10.3389/fphys.2020.601237

**Published:** 2020-12-15

**Authors:** Maycon I. O. Milanez, Amanda C. Veiga, Beatriz S. Martins, Roberto B. Pontes, Cassia T. Bergamaschi, Ruy R. Campos, Erika E. Nishi

**Affiliations:** Department of Physiology, Cardiovascular Division, Escola Paulista de Medicina, Universidade Federal de São Paulo, São Paulo, Brazil

**Keywords:** renal denervation, neuroinflammation, γ-aminobutyric acid, renovascular hypertension, afferent innervation

## Abstract

Renal sensory activity is centrally integrated within brain nuclei involved in the control of cardiovascular function, suggesting that renal afferents regulate basal and reflex sympathetic vasomotor activity. Evidence has shown that renal deafferentation (DAx) evokes a hypotensive and sympathoinhibitory effect in experimental models of cardiovascular diseases; however, the underlying mechanisms involved in this phenomenon need to be clarified, especially those related to central aspects. We aimed to investigate the role of renal afferents in the control of γ-aminobutyric acid (GABA)ergic inputs to the paraventricular nucleus (PVN) of the hypothalamus in renovascular hypertensive (2K1C) rats and their influence in the regulation of cardiovascular function. Hypertension was induced by clipping the left renal artery. After 4 weeks, renal DAx was performed by exposing the left renal nerve to a 33 mM capsaicin solution for 15 min. After 2 weeks of DAx, microinjection of muscimol into the PVN was performed in order to evaluate the influence of GABAergic activity in the PVN and its contribution to the control of renal sympathetic nerve activity (rSNA) and blood pressure (BP). Muscimol microinjected into the PVN triggered a higher drop in BP and rSNA in the 2K1C rats and renal DAx mitigated these responses. These results suggest that renal afferents are involved in the GABAergic changes found in the PVN of 2K1C rats. Although the functional significance of this phenomenon needs to be clarified, it is reasonable to speculate that GABAergic alterations occur to mitigate microglia activation-induced sympathoexcitation in the PVN of 2K1C rats.

## Introduction

Elevated sympathetic vasomotor activity is implicated in cardiovascular diseases, either experimentally or clinically (Grassi, 2010; Campos et al., 2015). Studies indicate that approaches aimed at reducing sympathetic vasomotor tone are beneficial in these conditions (Nishi et al., 2019; Lopes et al., 2020; Veiga et al., 2020); therefore, the underlying mechanisms by which such benefits occur have been extensively studied in recent years, especially how the central nervous system (CNS) drives sympathetic vasomotor overactivation in pathophysiological conditions (Oliveira-Sales et al., 2009; Nishi et al., 2019).

It is well-described that phenotypic changes in brain nuclei involved in cardiovascular control contribute to the maintenance of high sympathetic outflow in cardiovascular diseases, such as hypertension or heart failure (Oliveira-Sales et al., 2009; Carillo et al., 2012; Milanez et al., 2020). In this context, hypotensive and sympathoinhibitory effects triggered by the pharmacological blockade of glutamatergic and angiotensinergic pathways in the CNS have been shown in cardiovascular diseases (Bergamaschi et al., 1995; de Oliveira-Sales et al., 2010; Ramchandra et al., 2012; Milanez et al., 2018). In addition, changes in γ-aminobutyric acid (GABA)ergic pathways involved in the control of sympathetic vasomotor activity have also been reported in the Goldblatt model of hypertension, two-kidney, one-clip (2K1C), and chronic kidney disease, suggesting that neurotransmission of an inhibitory nature is also altered in the condition of cardiovascular disease (Biancardi et al., 2010; Nishihara et al., 2017; Milanez et al., 2020). Although it is not fully known what leads sympathetic activation in cardiovascular diseases, recent studies highlight the role of sensory signaling in these alterations, especially the renal sensory system (Nishihara et al., 2017; Zheng et al., 2018).

Currently, it is possible to study the selective role of sensory renal afferents and evaluate the effects evoked by their removal in the course of the disease (Foss et al., 2015). For example, we recently showed that the selective renal nerve deafferentation (DAx) causes a decrease in blood pressure (BP) and renal sympathetic nerve activity (rSNA) in the Goldblatt 2K1C rats and 5/6 nephrectomy model of chronic renal failure, indicating that the renal sensory afferents are centrally integrated and trigger changes in central nuclei involved in the control of cardiovascular function (Lopes et al., 2020; Veiga et al., 2020). In fact, evidences indicate that brain nuclei involved in the cardiovascular control, such as the rostral ventrolateral medulla (RVLM) and the paraventricular nucleus (PVN) of the hypothalamus, receive projections of sensory renal afferents (Nishi et al., 2017; Zheng and Patel, 2017).

In order to clarify the repercussions of renal denervation on neurotransmission in the PVN in mice with chronic renal failure, Nishihara et al. (2017) showed that the removal of total renal nerve, that is both sympathetic and sensory fibers, increased GABAergic activity in the PVN accompanied by a robust hypotensive effect. It is rational to speculate that the improvement in the GABAergic neurotransmission in the PVN was caused, in part, by the absence of renal sensory afferents; however, the underlying mechanisms need to be better explored. A previous study by our group showed that total renal denervation decreased oxidative stress within the RVLM and PVN independently of BP reduction in 2K1C rats (Nishi et al., 2019). However, these studies did not distinguish the specific role of sympathetic or sensory fibers from the kidneys mediating changes in CNS pathways.

Setiadi et al. (2020) showed that inflammation plays a role in maintaining hypertension in the Goldblatt model, as the treatment with pentoxifylline, a tumor necrosis factor-α (TNF-α) synthesis inhibitor, evoked a drop in BP and rSNA, as well as a reduction in neuronal activity in the PVN of 2K1C rats. In addition, evidence suggests that the central GABAergic system can attenuate the release of pro-inflammatory agents from microglia cells, playing an important anti-inflammatory role in the CNS (Lee et al., 2011). Therefore, it is reasonable to speculate that changes in the GABAergic inputs to the PVN triggered by the renovascular hypertension may contribute to inflammation-induced sympathoexcitation in the 2K1C rats.

Thus, considering that we previously reported a robust renal sympathoinhibitory effect caused by DAx in the 2K1C rats (Lopes et al., 2020), we hypothesized in the present study that this phenomenon may be triggered by alterations in the GABAergic inputs into the PVN of 2K1C DAx rats. In addition, in order to analyze the effects of DAx in terms of sympathetic vasomotor activity reflex control, we evaluated the cardiac baroreflex sensitivity in the presence and absence of renal sensory activity in 2K1C rats.

## Materials and Methods

### Animals and Experimental Protocol

All experimental procedures used in this study were conducted according to the guidelines recommended by the National Institutes of Health and were approved by the Ethics in Research Committee of the Escola Paulista de Medicina – Universidade Federal de São Paulo – (3629190314). Male Wistar rats were used at 5 weeks of age (150–180 g) for the induction of renovascular hypertension. In addition, male Wistar rats at 9 weeks of age (300–350 g) were used as the control group. The animals were housed in groups of 3–4 in standard polypropylene cages in a room maintained at 22 ± 1°C, humidity 60% and with a 12:12 h light-dark cycle (lights on at 7 am) and were allowed free access to food and water.

Thirty Wistar rats were distributed into three independent experimental groups: a control group underwent sham surgery for DAx (*n* = 10), a 2K1C group underwent sham surgery for DAx (*n* = 10), and a 2K1C group underwent DAx (2K1C DAx; *n* = 10). To induce renovascular hypertension, a silver clip (gap width 0.2 mm) was implanted around the left renal artery of rats anesthetized with an i.p. injection of ketamine and xylazine (100 and 10 mg/kg, respectively; Vetbrands, Jacareí, SP, Brazil). Age-matched rats were used as controls, which did not undergo clip implantation. After 4 weeks of renal artery clipping, unilateral DAx was performed by exposing the left or ischemic kidney nerve to 33 mM capsaicin (diluted in 0.1% ethanol and 0.1% Tween 80) for 15 min. In the sham groups, rats underwent the same surgical procedure described above, but with vehicle application (0.1% ethanol and 0.1% Tween 80) around the renal artery (Foss et al., 2015). As previously shown in another study by our group, the confirmation of DAx was verified by the reduction in calcitonin gene-related peptide (CGRP) expression by immunohistochemical analysis in the renal pelvis after 2 weeks of the procedure (Lopes et al., 2020) when all data were collected. Two independent sets of experiments were performed: one set for BP and rSNA responses triggered by muscimol, an agonist of GABA-A receptors, microinjected into PVN and another set for analysis of cardiac baroreflex sensitivity in conscious rats and microglia activation analysis in the PVN.

### Direct Measurement of Blood Pressure and Heart Rate and Baroreflex Sensitivity Evaluation in Conscious Rats

After 2 weeks of sham surgery or DAx, control (*n* = 5), 2K1C (*n* = 5) and 2K1C DAx (*n* = 5) rats were respectively anesthetized with ketamine and xylazine (100 and 10 mg/kg i.p., respectively), the femoral artery was catheterized for direct recording of arterial BP, and the femoral vein was catheterized for drug infusions. After surgical recovery (~24 h), pulsatile arterial pressure (PAP) was recorded in conscious rats using a BP signal amplifier (PowerLab System, ADInstruments, Sydney, Australia). The average values of baseline mean arterial pressure (MAP, mmHg), systolic arterial pressure (SAP, mmHg), diastolic arterial pressure (DAP, mmHg), and heart rate (HR, bpm) were obtained from the direct recording of pulsatile BP by 15-min continuous recording. MAP and HR changes to the various stimuli were expressed as the change (*Δ*) from the baseline value obtained immediately before each test (i.e., a basal record of 10 min).

Reflex bradycardia and tachycardia responses induced by bolus infusion of phenylephrine (3, 5, and 10 μg/rat, i.v.) and sodium nitroprusside (5, 12 e 20 μg/rat, i.v.; Sigma Aldrich Co, St. Louis, MO, United States) were measured, as previously reported (Lincevicius et al., 2017). Cardiac baroreflex sensitivity was evaluated by the mean index relating changes in HR to the changes in MAP and expressed as beats per mmHg (bpm/mmHg, baroreflex function sensitivity).

### Evaluation of Microglia Activation by Iba1-Positive Cells Analysis in the PVN by Immunohistochemistry

After deep anesthesia with ketamine and xylazine, control (*n* = 5), 2K1C (*n* = 5), and 2K1C DAx (*n* = 5) rats were perfused transcardially with 400 ml of 0.1 M buffered saline (pH 7.4), followed by 400 ml of 4% (w/v) paraformaldehyde (PFA). Brains were removed and post-fixed in 4% PFA for 4 h followed by cryoprotection in 20% (w/v) sucrose at 4°C until the brains sunk. Then, serial coronal slices at 30 μm were performed according to a brain atlas (1.8–2.10 mm from the bregma; Paxinos and Watson, 2007) in a cryostat. Immunodetection was performed by the free-floating method, and the sections were incubated with anti-Iba1 antibody (1:200, Abcam) overnight at room temperature. Subsequently, the sections were incubated in biotinylated secondary antibody (Jackson ImmunoResearch Laboratories, West Grove, PA, 1:500 dilution in 1% normal horse serum) for 24 h at room temperature. The sections were then incubated in ExtrAvidin-HRP (1:1,500, Sigma-Aldrich) for at least 4 h at room temperature. Subsequently, Iba1-positive cells were visualized using imidazole-intensified diaminobenzidine (DAB) reaction in which peroxide was generated by glucose oxidase. Then, the sections were mounted onto glass microscope slides with 0.5% gelatin and allowed to air-dry. Once dry, the slides were dehydrated in ethanol (70, 90, and 2 × 100%), cleared in xylol and cover-slipped in DPX mountant (Sigma-Aldrich). Tissue sections were inspected using an Eclipse 80i microscope (Nikon Instruments, Melville, NY) and a Nikon digital sight DSRi1 camera with NIS-Elements software. Images were uniformly adjusted for brightness and contrast and photographed under ideal magnification (20× objective) for high resolution photos. Blinded and stereological method for analysis was performed in at least three sections containing the PVN in every fourth section (each section 120 μm apart) that were averaged for each sample. Immunohistochemical control was performed in a set of sections for each group in which either the primary or secondary antibody was omitted. We verified no non-specific staining (data not shown).

### Analysis of the Renal Sympathetic Nerve Activity and Microinjection of Muscimol Into the PVN in Urethane-Anesthetized Rats

After recording the cardiovascular parameters in conscious rats, the animals were slowly anesthetized with urethane (1.2 g/kg, i.v.; Sigma-Aldrich Co., St. Louis, MO, United States) to avoid any change in baseline cardiovascular conditions. Tracheotomy was performed to reduce airway resistance. The nerve of the left or ischemic kidney was exposed retroperitoneally for the implantation of bipolar silver electrode, and activity was amplified (20 K) and filtered (100–1,000 Hz; NeuroLog, Digitimer, Welwyn Garden City, Herts, United Kingdom). The raw nerve signal was passed through a spike discriminator using Spike Histogram software (PowerLab, ADInstruments) to remove background noise, and the total nerve activity was expressed in spikes per second (spikes/s), as commonly used by previous studies that compared multifiber nerve recording among different groups (Wei and Felder, 2002; Oliveira-Sales et al., 2009; Veiga et al., 2020). The number of spikes/bursts reflects cardiovascular barosensitive fibers, as previously shown (Malpas and Ninomiya, 1992). The spikes/s changes to the various stimuli were expressed as the change (*Δ*) from the baseline value obtained immediately before each test (i.e., a basal record of 10 min).

**Figure 1:**
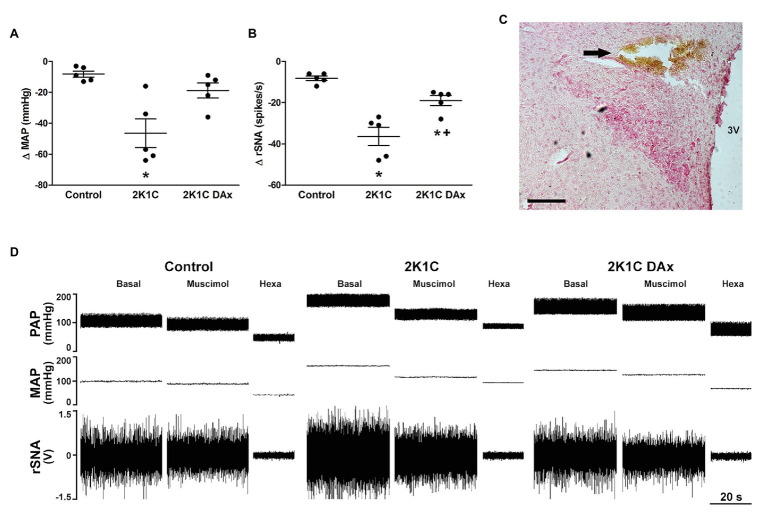
Maximum variation triggered by the microinjection of muscimol into paraventricular nucleus (PVN) of the hypothalamus in terms of **(A)** mean arterial pressure (MAP) and **(B)** renal sympathetic nerve activity (rSNA) in the control, hypertensive (2K1C) and renal deafferented hypertensive (2K1C DAx) rats. ^*^p < 0.05 in relation to the control group; ^+^*p* < 0.05 in relation to the 2K1C group (one-way ANOVA followed by Tukey’s *post hoc* test). **(C)** Representative histological image: the arrow indicates the site of microinjection into the PVN. Scale bar = 100 μm. 3 V, third ventricle. **(D)** Diagram representing the effect of muscimol microinjected into the PVN in terms of pulsatile arterial pressure (PAP), MAP, and rSNA in the control, hypertensive (2K1C) and renal deafferented hypertensive (2K1C DAx) rats. Microinjection of muscimol into the PVN triggered a higher drop in MAP and rSNA in 2K1C rats and the renal deafferentation blunted these findings. Hexa, hexamethonium.

Urethane-anesthetized rats were placed in a stereotaxic apparatus (David Kopf Instruments, Tujunga, CA) for the microinjection procedure. The bregma was exposed, and a craniotomy of approximately 3 × 3 mm was performed dorsally to the bregma with a drill. The PVN was located 1.8 mm caudal to bregma, 0.5 mm lateral to the midline, and 7.8 mm from dorsal surface (bite bar = 3.6 mm). Bilateral microinjections of muscimol (10 mM in 100 nl) into PVN were performed with the use of glass micropipettes with tip diameters of 10–20 μm connected to a nitrogen pressure injector (Channel pressure injector – MicroData Instruments Inc., United States), as previously reported (Oliveira-Sales et al., 2009; Carillo et al., 2012). At the end of the experiments, the background noise of rSNA was determined by hexamethonium bromide administration (30 mg/kg, i.v.) to select postganglionic sympathetic ongoing nerve activity, and the efficacies of the microinjections were confirmed by administering Evans Blue 2% (100 nl) into the PVN (Figure 1C). As previously reported, microinjection of 100 nl is capable of covering approximately 0.3–0.7 mm (spherical pattern of distribution) when applied to hypothalamic tissue (Segura et al., 1992).

### Statistical Analysis

Values are presented as the mean ± SEM. Data were analyzed by one-way ANOVA or repeated measures ANOVA followed by Tukey’s *post hoc* test was applied to compare data among control, 2K1C e 2K1C DAx groups. A value of *p* < 0.05 was considered statistically significant.

## Results

Basal values of SAP, DAP, MAP, and HR in the control, 2K1C and 2K1C DAx groups are exhibited in Table 1.

**Table 1:** Basal values of systolic arterial pressure (SAP), diastolic arterial pressure (DAP), MAP, and HR in the control, 2K1C and 2K1C DAx obtained from conscious rats.

	Control (*n* = 5)	2K1C (*n* = 5)	2K1C DAx (*n* = 5)
SAP (mmHg)	129 ± 4	228 ± 10^*^	198 ± 6^*^^+^
DAP (mmHg)	91 ± 2	160 ± 8^*^	133 ± 3^*^ ^+^
MAP (mmHg)	104 ± 2	183 ± 8^*^	154 ± 4^*^ ^+^
HR (bpm)	379 ± 12	412 ± 9	378 ± 9

* *p* < 0.05 in relation to the control group.

+ *p* < 0.05 in relation to the 2K1C group (one-way ANOVA followed by Tukey’s *post hoc* test).

### Cardiovascular and Renal Sympathetic Vasomotor Responses Evoked by Muscimol Injection Into the PVN

Muscimol microinjected into the PVN triggered a more intense MAP variation (*Δ*) in the 2K1C rats (CTL vs. 2K1C: −8 ± 2 vs. −46 ± 9^*^ ΔmmHg, *p* < 0.05; Figure 1A). In addition, the magnitude of rSNA reduction was higher in 2K1C rats (CTL vs. 2K1C: −8 ± 1 vs. −36 ± 4^*^ Δspikes/s, *p* < 0.05). DAx attenuated the MAP, and the renal sympathoinhibitory responses evoked by muscimol microinjected into the PVN when compared to 2K1C (2K1C vs. 2K1C DAx: −36 ± 4 vs. −19 ± 2^*^ Δspikes/s, *p* < 0.05; Figure 1B). The representative diagram related to these responses is exhibited in Figure 1D.

### Effects of Selective Renal Nerve Deafferentation on Cardiac Baroreflex Sensitivity

**Figure 2:**
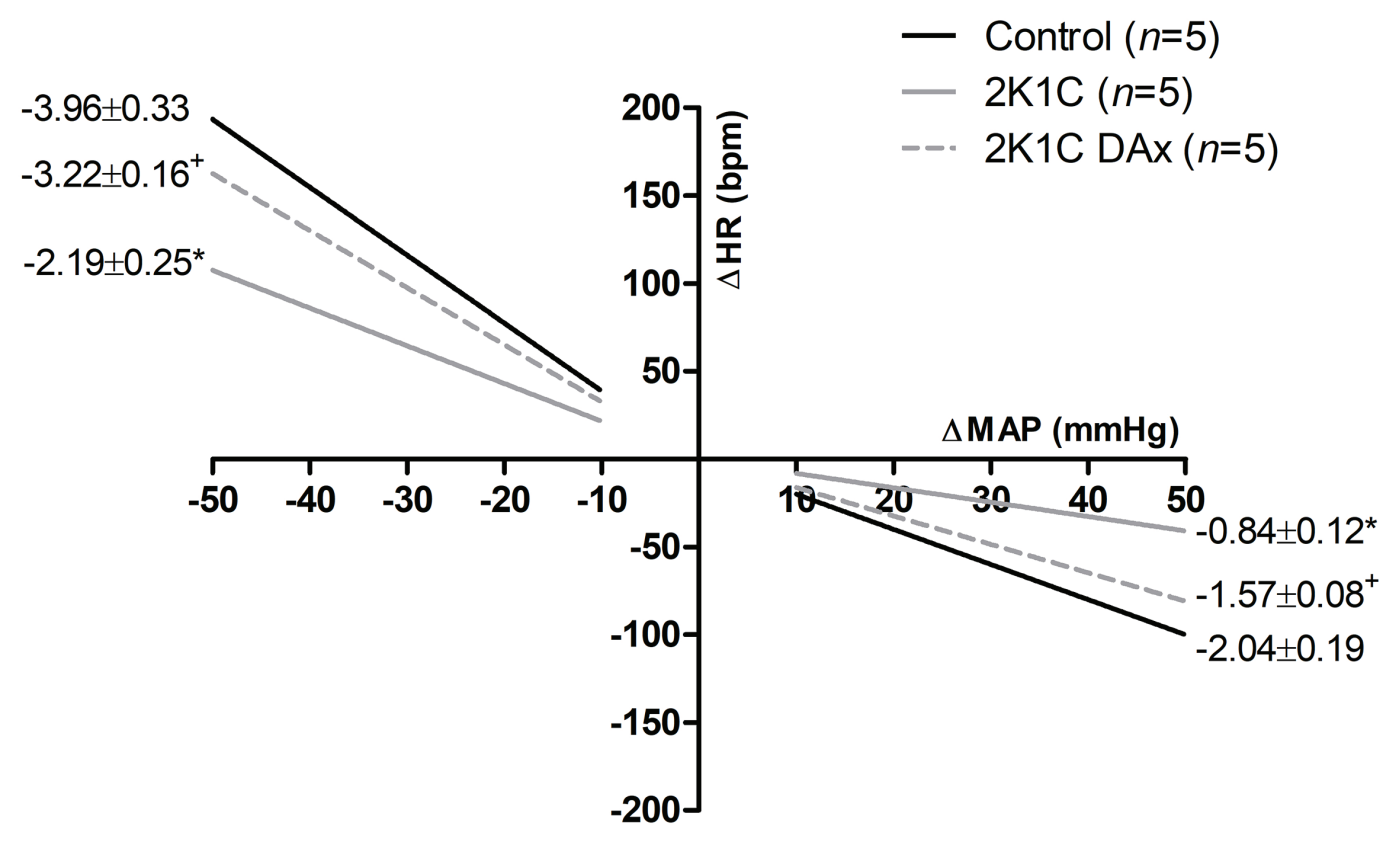
Heart rate (HR) reflex responses induced by unloading or loading of arterial baroreceptors in the control, 2K1C, and 2K1C DAx rats. ^*^p < 0.05 in relation to the control group; ^+^*p* < 0.05 in relation to the 2K1C group (repeated measures ANOVA followed by Tukey’s *post hoc* test).

Figure 2 shows the effects of DAx on cardiac baroreflex sensitivity in 2K1C rats. We found that the induction of renovascular hypertension caused an impairment in this parameter and that selective renal deafferentation normalized the cardiac baroreflex response, both for tachycardia and bradycardia responses.

### Effects of Selective Renal Nerve Deafferentation on Iba1-Positive Cells in the PVN of 2K1C Rats

**Figure 3:**
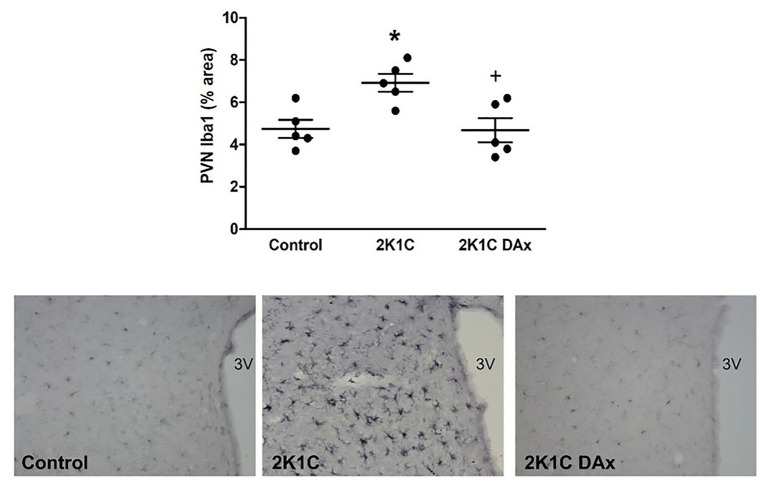
Analysis of ionized calcium binding adaptor molecule 1 (Iba1)-positive cells in the PVN of the hypothalamus in the control, 2K1C, and 2K1C DAx rats **(upper panel)**. ^*^p < 0.05 in relation to the control group; ^+^*p* < 0.05 in relation to the 2K1C group (one-way ANOVA followed by Tukey’s *post hoc* test). Representative histological section of Iba1 analysis among groups **(lower panel)**. Scale bar = 100 μm. 3 V: third ventricle.

Immunohistochemistry analysis revealed that the induction of renovascular hypertension caused an increase in Iba1-positive cells in the PVN (control vs. 2K1C: 4.7 ± 0.4 vs. 7 ± 0.4^*^, *p* < 0.05). However, DAx normalized this parameter in 2K1C rats (2K1C vs. 2K1C DAx: 7 ± 0.4 vs. 4.7 ± 0.5^*^, *p* < 0.05; Figure 3).

## Discussion

The main findings of this study demonstrate that changes in renal sensory activity in 2K1C rats evoke an increase in the GABAergic inputs into the PVN and contribute to impaired cardiac baroreflex sensitivity. In addition, renal afferent activity may be involved in the induction of inflammatory state in the PVN of 2K1C rats.

Although it has been shown that RVLM is an important site integrating renal afferent signaling (Nishi et al., 2017), glutamatergic neurons in the PVN that project into RVLM are activated by stimulation of renal afferents (Xu et al., 2015). We found that stimulation of GABAergic neurons with muscimol microinjected into the PVN of 2K1C rats produced a greater drop in BP and rSNA, suggesting that GABAergic inputs to the PVN may be increased in renovascular hypertension. In fact, increased GABAergic terminal density within the PVN in Goldblatt rats was reported (Biancardi et al., 2010). Similarly, bicuculline, a GABAergic antagonist, intrathecally administered triggered a greater increase in BP and rSNA in renovascular hypertensive rats, indicating that the spinal GABAergic system involved in the control of sympathetic vasomotor activity is also altered in the model (Milanez et al., 2020). In addition, previous studies carried out on renal-wrapped hypertensive rats – another experimental model of renovascular hypertension – reported that, despite a reduction in GABAergic inputs to the PVN in the onset of hypertension, there is a higher GABAergic activity in the PVN in the chronic phase of hypertension (Martin and Haywood, 1998; Haywood et al., 2001). These findings suggest that a higher inhibitory input to CNS regions involved in the control of cardiovascular function act protectively, mitigating an exacerbation of sympathetic vasomotor over activity in the 2K1C model. In fact, both GABAergic and glutamatergic innervation densities are augmented in the PVN of renovascular hypertensive rats (Biancardi et al., 2010). Thus, an imbalance between excitatory and inhibitory inputs to sympathetic premotor neurons leads to increased sympathetic drive in renovascular hypertension.

It is noteworthy that GABA regulates the activity of microglia cells through activation of its Type A receptors; Lee et al. (2011) showed that the release of TNF-α and interleukin-6 induced by lipopolysaccharide from microglia cells is blunted by muscimol, suggesting an anti-inflammatory action of the central GABAergic system. Thus, we cannot rule out the hypothesis that an increase in GABAergic inputs to the PVN occurs in order to mitigate the local inflammatory state and reduce the sympathoexcitation in renovascular hypertension (Wu et al., 2012). This speculation can be strengthened by the fact that the cardiovascular responses evoked by the muscimol microinjected into the PVN were normalized in the 2K1C DAx rats, as well as the Iba-1 percentage area in the PVN.

Nishihara et al. (2017) found a reduction in the sympathoexcitatory effects triggered by the microinjection of bicuculline into the PVN in mice with chronic renal failure, indicating that GABAergic inputs to PVN is reduced in this condition. Interestingly, the authors showed that total renal denervation was able to normalize the sympathetic responses evoked by GABAergic antagonism in the PVN in the chronic renal failure (Nishihara et al., 2017). Such evidence, together with the findings of the present study, brings to light an important aspect regarding the central changes involved in the genesis/maintenance of high sympathetic vasomotor activity, that is, although the sympathoexcitation is a characteristic commonly found in cardiorenal diseases, the neural mechanisms that culminate in this anomaly can be distinct.

In previous studies, we showed that DAx of the ischemic kidney reduced BP partially and decreased sympathetic hyperactivity to both the ischemic and contralateral kidneys (Lopes et al., 2020) and that the total renal denervation improved cardiac and renal, but not lumbar, baroreflex sensitivity in 2K1C rats (Lincevicius et al., 2017). Interestingly, in the present study, we show that renal DAx improves the reflex control of HR by arterial baroreceptors in the 2K1C model, potentially due to the modulatory influence of renal sensory fibers that project to regions involved in the control of baroreflex function, such as the nucleus tractus solitarii, PVN, and RVLM, differently influencing the control of sympathetic vasomotor outflow (Solano-Flores et al., 1997; Xu et al., 2015; Nishi et al., 2017). Moreover, this study suggests that the GABAergic inputs to the PVN may exert a tonic inhibitory action on the local neurons involved in controlling the baroreflex function in the 2K1C model, contributing to an impairment in its sensitivity. However, we cannot rule out the possibility that the improvement in the baroreflex control of cardiac function may have occurred by improving the brain oxidative system and/or mitigating the inflammatory process caused by the removal of renal sensory activity (Oliveira-Sales et al., 2009; Fonkoue et al., 2019). In addition to GABA, functional changes in other neurotransmitters/neuromodulators contributing to the impairment in the baroreflex sensitivity have been described in the 2K1C rats; for example, a higher spinal angiotensinergic and glutamatergic activity in the Goldblatt model blunt the reflex control of rSNA by arterial baroreceptors (Milanez et al., 2018). Although not yet fully clarified, the role of baroreflex in the long-term control of BP cannot be ruled out (Thrasher, 2006). Therefore, it is reasonable to assume that part of the hypotensive effect triggered by renal DAx in 2K1C rats was evoked by the improvement in baroreflex control of cardiac function (Tsyrlin et al., 2013). Another possible mechanism by which DAx triggers BP reduction is attenuating intrarenal angiotensinergic activity in the 2K1C model (Lopes et al., 2020).

Several studies have shown that oxidative imbalance and inflammation in the brain contribute to sympathoexcitation in neurogenic hypertension, especially in medullary and hypothalamic sites. Previous studies showed that oxidative stress induced by angiotensin II-NADPH oxidase pathway in the RVLM and PVN plays a critical role in the development of hypertension in the 2K1C model (Oliveira-Sales et al., 2009; de Oliveira-Sales et al., 2010), and total renal denervation was capable of reversing it (Nishi et al., 2019). In the present study, we show greater activation of microglia in the PVN that was reversed by renal DAx in the 2K1C rats. A previous study related the development of neurogenic hypertension induced by systemic inflammation with increased microglial activity and the generation of reactive oxygen species in the RVLM, given by the increase in the subunits of NADPH oxidase and superoxide anion that contributed to the maintenance of higher BP (Wu et al., 2012). Moreover, angiotensin II-induced microglial activation and hypertension in the PVN were reversed by intracerebroventricular infusion of minocycline, an anti-inflammatory antibiotic, and another study showed that total renal denervation decreased microglial activation and reactive oxygen species in the cortex, white matter, and PVN of stroke prone spontaneously hypertensive rats (SHRSP; Shi et al., 2010; Nakagawa et al., 2013). In accordance with these studies, we show that afferent signaling from the ischemic kidney may contribute to neuroinflammation-induced sympathetic vasomotor overactivity in the PVN of 2K1C rats. However, the underlying mechanisms by which renal DAx attenuated glial activity in the PVN of 2K1C rats need to be further explored, as well as whether this phenomenon is related to the autonomic and cardiovascular effects found in this study.

In the present study, we assessed the inflammatory state of PVN indirectly through the analysis of Iba-1. Although it is a marker widely used for this purpose, it is necessary to evaluate, locally, and systemically, the inflammatory profile through the quantification of pro-inflammatory agents, such as TNF-α and other cytokines. Moreover, BP measurement was performed during the daylight, at the end of the dark cycle. Thus, circadian rhythms and food intake may influence to some degree of the basal level of BP. Although all animals underwent the same experimental protocol, it would be necessary to measure cardiovascular parameters by telemetry over the whole period to detect the influences of light and dark cycles during the course of 2K1C hypertension.

Taken together, these results suggest that renal afferent activity is involved in the changes of the neuronal inputs to the PVN of 2K1C rats, especially those inputs related to inhibitory activity. Although the functional significance of this finding still needs to be clarified, it is likely that the increase in GABAergic inputs to PVN occurs in order to mitigate a higher sympathoexcitation in renovascular hypertension. Moreover, we found that the impairment in baroreflex control of HR in 2K1C rats is mediated, in part, by alterations in the renal sensory system.

## Data Availability Statement

The raw data supporting the conclusions of this article will be made available by the authors, without undue reservation.

## Ethics Statement

The animal study was reviewed and approved by Ethics in Research Committee of the Escola Paulista de Medicina – Universidade Federal de São Paulo – (3629190314).

## Author Contributions

EN, CB, and RC: conception or design of the work. MM, AV, BM, and RP: acquisition, analysis, and interpretation of data for the work. MM and AV: drafting the work. All authors revised the manuscript critically for important intellectual content, approved the final version of the manuscript, agreed to be accountable for all aspects of the work in ensuring that questions related to the accuracy or integrity of any part of the work are appropriately investigated and resolved. All persons designated as authors qualify for authorship. All those who qualify for authorship are listed.

### Conflict of Interest

The authors declare that the research was conducted in the absence of any commercial or financial relationships that could be construed as a potential conflict of interest.

## References

[ref1] Bergamaschi C. Campos R. R. Schor N. Lopes O. U. (1995). Role of the rostral ventrolateral medulla in maintenance of blood pressure in rats with Goldblatt hypertension. Hypertension 26, 1117–1120. 10.1161/01.HYP.26.6.1117, PMID: 7498979

[ref2] Biancardi V. C. Campos R. R. Stern J. E. (2010). Altered balance of gamma-aminobutyricacidergic and glutamatergic afferent inputs in rostral ventrolateral medulla-projecting neurons in the paraventricular nucleus of the hypothalamus of renovascular hypertensive rats. J. Comp. Neurol. 518, 567–585. 10.1002/cne.22256, PMID: 20034060 PMC4428175

[ref3] Campos R. R. Oliveira-Sales E. B. Nishi E. E. Paton J. F. Bergamaschi C. T. (2015). Mechanisms of renal sympathetic activation in renovascular hypertension. Exp. Physiol. 100, 496–501. 10.1113/expphysiol.2014.079855, PMID: 25639235

[ref4] Carillo B. A. Oliveira-Sales E. B. Andersen M. Tufik S. Hipolide D. Santos A. A.. (2012). Changes in GABAergic inputs in the paraventricular nucleus maintain sympathetic vasomotor tone in chronic heart failure. Auton. Neurosci. 171, 41–48. 10.1016/j.autneu.2012.10.005, PMID: 23146621

[ref5] de Oliveira-Sales E. B. Nishi E. E. Boim M. A. Dolnikoff M. S. Bergamaschi C. T. Campos R. R. (2010). Upregulation of AT1R and iNOS in the rostral ventrolateral medulla (RVLM) is essential for the sympathetic hyperactivity and hypertension in the 2K-1C wistar rat model. Am. J. Hypertens. 23, 708–715. 10.1038/ajh.2010.64, PMID: 20360752

[ref6] Fonkoue I. T. Le N. A. Kankam M. L. DaCosta D. Jones T. N. Marvar P. J.. (2019). Sympathoexcitation and impaired arterial baroreflex sensitivity are linked to vascular inflammation in individuals with elevated resting blood pressure. Phys. Rep. 7:e14057. 10.14814/phy2.14057, PMID: 30968587 PMC6456445

[ref7] Foss J. D. Wainford R. D. Engeland W. C. Fink G. D. Osborn J. W. (2015). A novel method of selective ablation of afferent renal nerves by periaxonal application of capsaicin. Am. J. Phys. Regul. Integr. Comp. Phys. 308, R112–R122. 10.1152/ajpregu.00427.2014, PMID: 25411365 PMC4297859

[ref8] Grassi G. (2010). Sympathetic neural activity in hypertension and related diseases. Am. J. Hypertens. 23, 1052–1060. 10.1038/ajh.2010.154, PMID: 20651696

[ref9] Haywood J. R. Mifflin S. W. Craig T. Calderon A. Hensler J. G. Hinojosa-Laborde C. (2001). Gamma-aminobutyric acid (GABA)--a function and binding in the paraventricular nucleus of the hypothalamus in chronic renal-wrap hypertension. Hypertension 37, 614–618. 10.1161/01.hyp.37.2.614, PMID: 11230344

[ref10] Lee M. Schwab C. McGeer P. L. (2011). Astrocytes are GABAergic cells that modulate microglial activity. Glia 59, 152–165. 10.1002/glia.21087, PMID: 21046567

[ref11] Lincevicius G. S. Shimoura C. G. Nishi E. E. Oliveira T. Cespedes J. G. Bergamaschi C. T.. (2017). Differential effects of renal denervation on arterial baroreceptor function in Goldblatt hypertension model. Auton. Neurosci. 208, 43–50. 10.1016/j.autneu.2017.06.002, PMID: 28688830

[ref12] Lopes N. R. Milanez M. I. O. Martins B. S. Veiga A. C. Ferreira G. R. Gomes G. N.. (2020). Afferent innervation of the ischemic kidney contributes to renal dysfunction in renovascular hypertensive rats. Pflugers Arch. 472, 325–334. 10.1007/s00424-019-02346-4, PMID: 31925527

[ref13] Malpas S. C. Ninomiya I. (1992). The amplitude and periodicity of synchronized renal sympathetic nerve discharges in anesthetized cats: differential effect of baroreceptor activity. J. Auton. Nerv. Syst. 40, 189–198. 10.1016/0165-1838(92)90200-z, PMID: 1460232

[ref14] Martin D. S. Haywood J. R. (1998). Reduced GABA inhibition of sympathetic function in renal-wrapped hypertensive rats. Am. J. Physiol. 275, R1523–R1529. 10.1152/ajpregu.1998.275.5.R1523, PMID: 9791069

[ref15] Milanez M. I. O. Nishi E. E. Rocha A. A. Bergamaschi C. T. Campos R. R. (2020). Interaction between angiotensin II and GABA in the spinal cord regulates sympathetic vasomotor activity in Goldblatt hypertension. Neurosci. Lett. 728:134976. 10.1016/j.neulet.2020.134976, PMID: 32304717

[ref16] Milanez M. I. O. Nishi É. Sato A. Y. S. Neto H. A. F. Bergamaschi C. T. Campos R. R. (2018). Control of renal sympathetic nerve activity by neurotransmitters in the spinal cord in Goldblatt hypertension. Brain Res. 1698, 43–53. 10.1016/j.brainres.2018.06.025, PMID: 29935157

[ref17] Nakagawa T. Hasegawa Y. Uekawa K. Ma M. Katayama T. Sueta D.. (2013). Renal denervation prevents stroke and brain injury via attenuation of oxidative stress in hypertensive rats. J. Am. Heart Assoc. 2:e000375. 10.1161/JAHA.113.000375, PMID: 24125845 PMC3835247

[ref20] Nishi E. E. Lopes N. R. Gomes G. N. Perry J. C. Sato A. Y. S. Naffah-Mazzacoratti M. G.. (2019). Renal denervation reduces sympathetic overactivation, brain oxidative stress, and renal injury in rats with renovascular hypertension independent of its effects on reducing blood pressure. Hypertens. Res. 42, 628–640. 10.1038/s41440-018-0171-9, PMID: 30573809

[ref21] Nishi E. E. Martins B. S. Milanez M. I. Lopes N. R. de Melo J. F. Pontes R. B.. (2017). Stimulation of renal afferent fibers leads to activation of catecholaminergic and non-catecholaminergic neurons in the medulla oblongata. Auton. Neurosci. 204, 48–56. 10.1016/j.autneu.2017.01.003, PMID: 28126464

[ref23] Nishihara M. Takesue K. Hirooka Y. (2017). Renal denervation enhances GABAergic input into the PVN leading to blood pressure lowering in chronic kidney disease. Auton. Neurosci. 204, 88–97. 10.1016/j.autneu.2016.09.018, PMID: 27729205

[ref24] Oliveira-Sales E. B. Nishi E. E. Carillo B. A. Boim M. A. Dolnikoff M. S. Bergamaschi C. T.. (2009). Oxidative stress in the sympathetic premotor neurons contributes to sympathetic activation in renovascular hypertension. Am. J. Hypertens. 22, 484–492. 10.1038/ajh.2009.17, PMID: 19229193

[ref25] Paxinos G. Watson C. (2007). The rat brain in stereotaxic coordinates. 6th Edn. London: Elsevier Academic Press.

[ref26] Ramchandra R. Hood S. G. Watson A. M. Allen A. M. May C. N. (2012). Central angiotensin type 1 receptor blockade decreases cardiac but not renal sympathetic nerve activity in heart failure. Hypertension 59, 634–641. 10.1161/HYPERTENSIONAHA.111.181131, PMID: 22311902 PMC3305814

[ref27] Segura T. Martin D. S. Sheridan P. J. Haywood J. R. (1992). Measurement of the distribution of [3H]bicuculline microinjected into the rat hypothalamus. J. Neurosci. Methods 41, 175–186. 10.1016/0165-0270(92)90059-M, PMID: 1564952

[ref28] Setiadi A. Korim W. S. May C. N. Yao S. T. (2020). Systemic administration of pentoxifylline attenuates the development of hypertension in renovascular hypertensive rats. Hypertens. Res. 43, 667–678. 10.1038/s41440-020-0412-6, PMID: 32060380

[ref29] Shi P. Diez-Freire C. Jun J. Y. Qi Y. Katovich M. J. Li Q.. (2010). Brain microglial cytokines in neurogenic hypertension. Hypertension 56, 297–303. 10.1161/HYPERTENSIONAHA.110.150409, PMID: 20547972 PMC2929640

[ref30] Solano-Flores L. P. Rosas-Arellano M. P. Ciriello J. (1997). Fos induction in central structures after afferent renal nerve stimulation. Brain Res. 753, 102–119. 10.1016/S0006-8993(96)01497-7, PMID: 9125437

[ref31] Thrasher T. N. (2006). Arterial baroreceptor input contributes to long-term control of blood pressure. Curr. Hypertens. Rep. 8, 249–254. 10.1007/s11906-006-0058-z, PMID: 17147924

[ref32] Tsyrlin V. A. Galagudza M. M. Kuzmenko N. V. Pliss M. G. Rubanova N. S. Shcherbin Y. I. (2013). Arterial baroreceptor reflex counteracts long-term blood pressure increase in the rat model of renovascular hypertension. PLoS One 8:e64788. 10.1371/journal.pone.0064788, PMID: 23762254 PMC3675197

[ref33] Veiga A. C. Milanez M. I. O. Ferreira G. R. Lopes N. R. Santos C. P. De Angelis K.. (2020). Selective afferent renal denervation mitigates renal and splanchnic sympathetic nerve overactivity and renal function in chronic kidney disease-induced hypertension. J. Hypertens. 38, 765–773. 10.1097/HJH.0000000000002304, PMID: 31764582

[ref34] Wei S. G. Felder R. B. (2002). Forebrain renin-angiotensin system has a tonic excitatory influence on renal sympathetic nerve activity. Am. J. Physiol. Heart Circ. Physiol. 282, H890–H895. 10.1152/ajpheart.2002.282.3.H890, PMID: 11834483

[ref35] Wu K. L. Chan S. H. Chan J. Y. (2012). Neuroinflammation and oxidative stress in rostral ventrolateral medulla contribute to neurogenic hypertension induced by systemic inflammation. J. Neuroinflammation 9:212. 10.1186/1742-2094-9-212, PMID: 22958438 PMC3462714

[ref36] Xu B. Zheng H. Liu X. Patel K. P. (2015). Activation of afferent renal nerves modulates RVLM-projecting PVN neurons. Am. J. Physiol. Heart Circ. Physiol. 308, H1103–H1111. 10.1152/ajpheart.00862.2014, PMID: 25637549 PMC4551125

[ref37] Zheng H. Katsurada K. Liu X. Knuepfer M. M. Patel K. P. (2018). Specific afferent renal denervation prevents reduction in neuronal nitric oxide synthase within the paraventricular nucleus in rats with chronic heart failure. Hypertension 72, 667–675. 10.1161/HYPERTENSIONAHA.118.11071, PMID: 30012866 PMC6202134

[ref38] Zheng H. Patel K. P. (2017). Integration of renal sensory afferents at the level of the paraventricular nucleus dictating sympathetic outflow. Auton. Neurosci. 204, 57–64. 10.1016/j.autneu.2016.08.008, PMID: 27527558 PMC5293680

